# Exploring the link between physical activity and professional quality of life among nurses: a cross-sectional study in Qatar

**DOI:** 10.1186/s12912-025-02967-4

**Published:** 2025-03-26

**Authors:** Kamaruddeen Mannethodi, George V. Joy, Kalpana Singh, Ederlie E. Pitiquen, Nabila Chaabna, Jibin Kunjavara, Abdulqadir J. Nashwan

**Affiliations:** Nursing and Midwifery Research Department, Hamad Medical Corporation, P.O. Box 3050, Doha, Qatar

**Keywords:** Physical activity, Professional quality of life, Nurses, Occupational health, Workplace well-being

## Abstract

**Background:**

Physical activity (PA) is crucial for maintaining physical and mental health, particularly for healthcare professionals like nurses, whose demanding roles can impact their well-being. Professional Quality of Life (ProQOL) reflects caregiving roles’ positive and negative outcomes, encompassing compassion, satisfaction, burnout, and trauma stress. This study explores the association between physical activity and ProQOL among nurses in Qatar.

**Methods:**

A quantitative, analytical, cross-sectional study was conducted among nurses working at Hamad Medical Corporation (HMC), with a sample size of 384, selected from approximately 10,000 nurses. Data was collected via an online survey using validated tools: the International Physical Activity Questionnaire-Short Form (IPAQ-SF) and the Professional Quality of Life Scale (ProQOL-5). Descriptive statistics, chi-square tests, and ANOVA were applied to analyze associations between PA levels, ProQOL, and various sociodemographic, occupational, and health-related factors.

**Results:**

A total of 430 nurses participated (82.1% female, mean age: 43.3 ± 7.2 years). Most participants reported engaging in low (46.5%) or moderate (51.6%) physical activity levels. Moderate PA was significantly associated with higher ProQOL scores, particularly compassion satisfaction (40.3 ± 6.3), compared to low (36.9 ± 7.2) and high PA levels (38.7 ± 6.9). Burnout showed no significant association with PA levels. Sociodemographic factors, including age and shift patterns, were associated with PA, with moderate PA being more common among slightly older nurses and those working 8-hour shifts.

**Conclusion:**

Moderate physical activity positively associates with nurses’ professional quality of life, particularly by enhancing compassion satisfaction. While burnout did not show any significant relationship with PA, other factors like organizational support and work environment may play a more pivotal role. Promoting moderate PA among nurses through workplace interventions and supportive policies could enhance their well-being and caregiving capacity.

**Clinical trial number:**

Not applicable.

## Introduction

Physical activity, an essential determinant of health, plays a crucial role in enhancing mental and physical well-being, yet remains insufficiently practiced by many, including healthcare professionals like nurses who are at the forefront of patient care [1]. Physical activity encompasses all forms of movement produced by skeletal muscles that require energy expenditure, and it can occur across multiple domains: leisure, occupation, education, home, and transport. Integrating physical activity across these domains helps meet recommended activity levels [2]. It plays a crucial role in preventing non-communicable diseases and improving mental health, thereby enhancing overall quality of life [3].

In Qatar, the nursing workforce is a critical component of the healthcare system, primarily composed of expatriates from diverse regions such as Latin America, South Asia, Southeast Asia, and the Middle East. This diversity brings unique strengths and challenges, including cultural variability in communication and care delivery styles. Nurses in Qatar typically possess a bachelor’s degree as the minimum qualification, with a smaller proportion holding master’s or doctoral degrees. The workforce is stratified into various roles, including bedside nurses, nurse educators, and administrative positions, with bedside nursing constituting the majority. Most nurses work under Hamad Medical Corporation (HMC), the leading provider of secondary and tertiary healthcare in the country. Common challenges faced by nurses in Qatar include adapting to rotating shifts with night duties, managing long working hours, and addressing the physical and emotional demands of caregiving in a multicultural environment.

Global recommendations for physical activity by World Health Organization (WHO) say adults should engage in at least 150–300 min of moderate-intensity or 75–150 min of vigorous-intensity physical activity per week. This can include leisure activities such as sports participation, exercise training, recreational activities like walking or dancing, and occupational and household activities such as cleaning, gardening, and caregiving [3]. Physical activity plays a crucial role in promoting both physical and mental health, while its absence negatively impacts individual well-being and the overall economy. Lack of exercise increases the risk of non-communicable diseases (NCDs) and contributes to higher morbidity and mortality rates. Conversely, regular physical activity reduces the risk of NCDs such as cardiovascular disease, type 2 diabetes, and cancer and offers various health benefits, including delayed mortality [4].

Global data estimate that 27.5% of adults fail to meet recommended physical activity guidelines, exhibiting poor physical behaviors such as insufficient physical activity, prolonged sedentary behavior, and inadequate sleep, all associated with a heightened risk of morbidity and mortality [3]. In the Middle East and North Africa (MENA) region, the prevalence of adults meeting the WHO physical activity recommendations varies significantly, ranging from as low as 13.2% in Sudan to as high as 94.9% in Jordan [5]. Physical activity among the public in Qatar is relatively low, and the national STEPS survey reports that 41% of the study participants were obese, and more than 60% of adults in the age group 18–64 were not participating in any physical activity [6].

Nurses are integral to healthcare systems, serving as caregivers and key advocates for health promotion, including physical activity. Despite the active nature of their roles, many nurses do not meet the recommended levels of physical activity. Research indicates that while nurses are in a prime position to promote physical activity to patients, their participation in such behaviors is often limited due to long shifts, irregular work schedules, and fatigue [7]. This creates a paradox in which nurses, who play a critical role in advocating for physical well-being, may not adequately engage in such practices.

In Qatar, programs like the online physical fitness program and the female-only recreation club have been introduced in Hamad Medical Corporation to encourage healthcare workers to stay active. However, challenges persist. Studies suggest that a lack of time due to overtime, inadequate resources, fatigue, and outside commitments further diminish nurses’ ability to participate in regular physical activity [8]. During working hours, most nurses engage in low-intensity physical activities such as walking and standing, with night shift nurses being particularly prone to sedentary behavior [9, 10]. Outside of work, many healthcare professionals remain physically inactive during their leisure time [11], highlighting the need for more comprehensive and accessible wellness programs.

For nurses, physical activity is not just about physical health; it is significantly associated with their professional quality of life. Regular physical activity improves cardiovascular health by reducing systolic blood pressure and enhances mental well-being, contributing to a better quality of life [12]. Higher physical activity levels were associated with greater professional quality of life, increased compassion satisfaction, and reduced burnout [13, 14]. In the United States, a study demonstrated a clear relationship between the duration of physical activity and compassion satisfaction, as well as a significant correlation between daily step counts and lower levels of burnout among nurses [15]. Furthermore, the COVID-19 pandemic has been shown to negatively affect both physical activity levels and healthcare workers’ overall quality of life, underscoring the importance of maintaining physical activity even during challenging times [16].

Despite the robust global evidence connecting physical activity with enhanced professional quality of life (ProQOL) among nurses, there remains a significant research gap in the Middle Eastern context, particularly in Qatar. Existing studies predominantly focus on Western populations, where cultural norms, work environments, and public health strategies vary considerably from those in the Middle East [5]. Nurses in this region may experience different work-life balances, social expectations, and stressors, which could affect their physical activity levels and ProQOL [17, 18]. This gap emphasizes the need for context-specific research to understand how regional factors such as workplace demands, climate, and cultural attitudes toward physical activity influence nurses’ health and well-being. By addressing these gaps, we can tailor public health strategies and workplace wellness programs to the unique needs of nurses in Qatar and the wider Middle Eastern region.

## Methodology

### Study design

This study adopts an analytical, cross-sectional design aimed at assessing the nurses’ levels of physical activity (PA) and associated factors and comparing professional quality of life (ProQOL) across varying levels of PA. The study uses validated questionnaires to measure physical activity and ProQOL.

### Study population and setting

The target population for this study comprises all nurses working at Hamad Medical Corporation (HMC), which includes approximately 10,000 nurses. Given the accessible population, the sample size was calculated using the formula: 𝑛=𝑍^2^𝑝𝑞/𝑑^2^. Where = 1.96 (for a 95% confidence level), *p* = 50% (proportion of the population assumed to have the desired PA level), 𝑞 = 1 – 𝑝, 𝑑 = 0.05 (precision). The resulting sample size was calculated to be 384 participants.

### Inclusion and exclusion criteria

Inclusion Criteria: All registered nurses working at HMC during the data collection period.

Exclusion Criteria: Nurses who have resigned from the organization or opted not to participate in the survey.

### Study procedures

A structured questionnaire with an information sheet was distributed to all nurses through their official email addresses. The study was conducted over a five-month period, from December 2023 to April 2024, using an online survey administered through Microsoft Forms.

### Measurements

The questionnaire comprised four sections:

Demographic details and work-related characteristics: Age (in years), gender (male or female), marital status (single, married, divorced, or widowed), position (bedside nurses, nurse educators, specialists, or administrative staff based on their current roles), working hours (8-hour shifts or 12-hour shifts per day), shift duty (day-only shifts, rotating shifts with night duty, and rotating shifts without night duty), health-related information (self-reported chronic diseases and COVID-19 infection status), and usage of physical activity self-monitoring devices.

International Physical Activity Questionnaire-Short Form (IPAQ-SF): This validated tool measures the intensity and frequency of physical activity over the past seven days. Based on MET minutes per week, the scoring criteria classify physical activity levels as low, moderate, or high. Test-retest reliability indicated good stability and a high reliability (α < 0.80) [19].

Professional Quality of Life-5 (ProQOL-5): ProQOL-5 evaluates the positive and negative effects of working in a caregiving environment. It measures three subscales: compassion satisfaction, burnout, and compassion fatigue, using a 5-point Likert scale. The Compassion Fatigue scale demonstrated strong internal consistency in this study, with a reported Cronbach’s alpha reliability ranging between 0.84 and 0.90, consistent with prior research findings [20].

### Ethical considerations

This study was conducted in accordance with the ethical principles outlined in the Declaration of Helsinki. All procedures involving human participants were reviewed and approved by the Institutional Review Board (IRB) of Hamad Medical Corporation (HMC) (MRC-01-23-369). Participation was voluntary, and informed consent was implied through survey completion. Participants were provided with an information sheet explaining the study’s purpose, procedures, and their right to withdraw at any time without consequences. No identifiable data were collected to ensure confidentiality and anonymity. The study adhered to ethical guidelines for research involving human participants.

### Statistical analysis plan

Descriptive statistics, including frequencies, percentages, means, and standard deviations, were used to summarize socio-demographic, occupational, and health-related characteristics of the participants. The total physical activity score was calculated by summing the metabolic equivalent task (MET) minutes per week for walking, moderate activity, and vigorous activity based on the scoring protocol. Physical activity levels were classified as low, moderate, or high according to the total MET minutes achieved. For the Professional Quality of Life Scale (ProQOL-5), scores for the three subscales—compassion satisfaction, burnout, and secondary traumatic stress were calculated by summing the item scores for each subscale, with higher scores indicating stronger manifestations of the respective domains. The distribution of continuous variables was assessed for normality using the Shapiro-Wilk test and visual methods such as Q-Q plots. To check the association chi-square tests for categorical variables and independent t-test and one-way ANOVA for comparing means across PA levels, to identify significant differences. Pearson or Spearman correlations, as appropriate, were used to examine relationships between continuous variables. Statistical significance was set at *p* < 0.05, and all analyses were conducted using STATA 17.0.

## Results

### Participants characteristics

A total of 430 participants responded to the survey with an average age of 43.3 ± 7.2 years. Almost half of the respondents, 48.1%, came from Latin America, 24.4% from South Asia, 16.3% from Southeast Asia, 8.1% from the Middle East, and 3.0% from Africa. The mean height of participants was 161.4 ± 8.2 cm, while their mean weight was 76.2 ± 42.1Kg. Table 1 details the participant characteristics.

**Table 1:** Participants’ characteristics

Variables	Level	*N* (%)
N		430
Gender	Male	77 (17.9%)
	Female	353 (82.1%)
Marital status	Single	104 (24.2%)
	Married	284 (66.0%)
	Widowed	4 (0.9%)
	Divorced	38 (8.8%)
Education	Doctorate	3 (0.7%)
	Master’s degree	84 (19.5%)
	Bachelor’s degree	328 (76.3%)
	Associate’s degree/Diploma	15 (3.5%)
Regular Duty hours	12 h/ day	23 (5.3%)
	8 h/day	407 (94.7%)
Type of duty	Day duty only	144 (33.5%)
	Rotating shift with night duty	272 (63.3%)
	Rotating shift without night duty	14 (3.3%)
Position	Bedside Nurses	349(81.16%)
	Educator & specialist	39(9.06%)
	Administration	42(9.76%)
Are you infected with covid-19?	No	198 (46.0%)
	Yes	232 (54.0%)
Physical activity level affected after COVID-19 infection	No	131 (56.5%)
	Yes, I feel easily exhausted after an infection	101(43.5%)
Do you feel that the current level of your physical activity is adequate for the main	No, I feel I need to do more for my physical health	15 (3.5%)
	Yes, it meets my needs for physical health	5 (1.2%)
How would you describe your diet?	Balanced and healthy;	8 (1.9%)
	balanced and healthy; high protein;	1 (0.2%)
	balanced and healthy; low carb;	2 (0.5%)
	high protein; other;	1 (0.2%)
	low carb; other;	1 (0.2%)
	other;	7 (1.6%)

### Physical activity levels and professional quality of life indicators

**Fig. 1:**
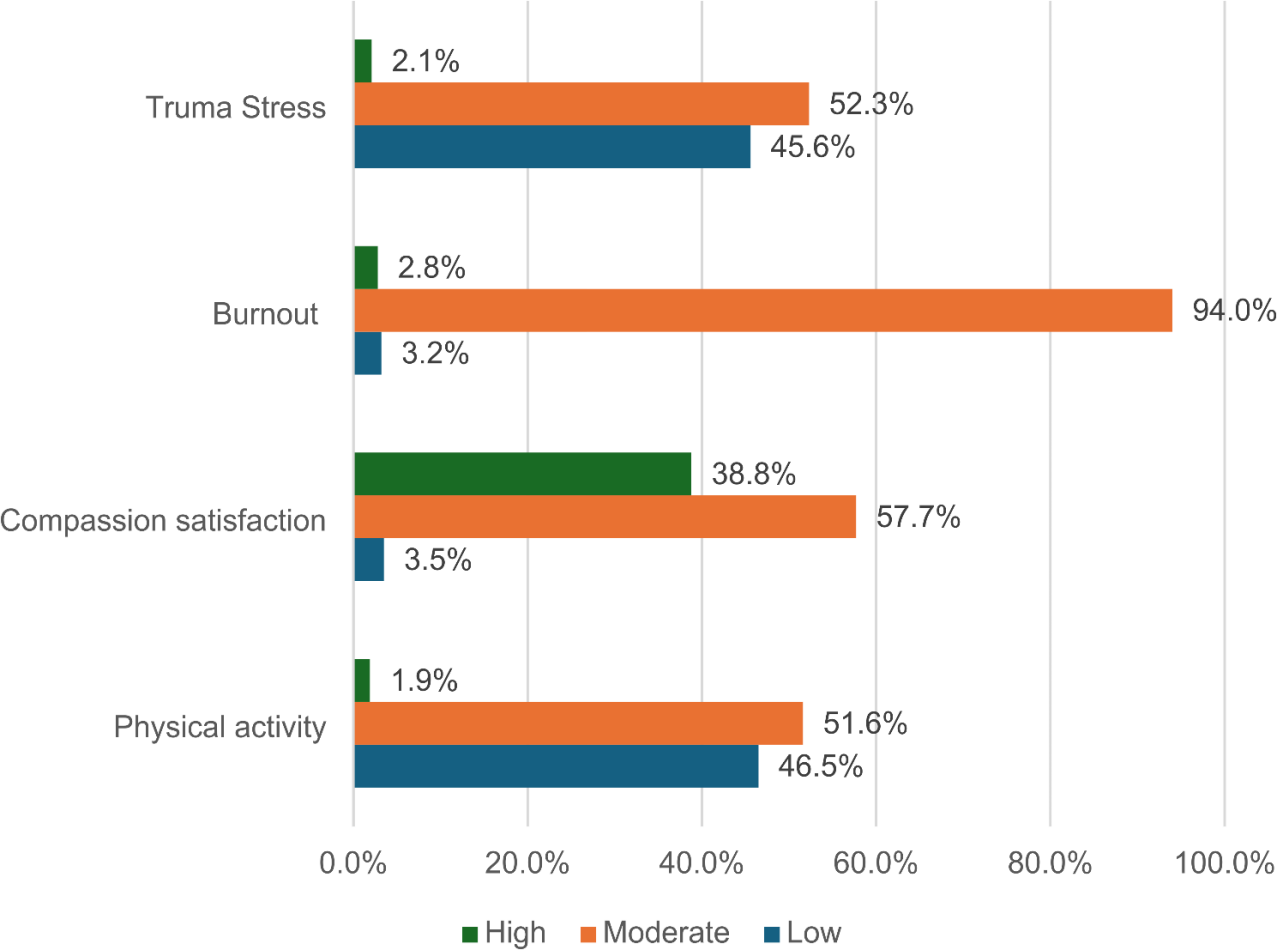
Physical activity and professional quality of life among nurses

Participants’ physical activity levels were categorized based on the IPAQ7 guideline: low, moderate, and high [19]. Most participants reported a low or medium level of physical activity (98.1%). Regarding professional quality of life indicators, compassion satisfaction was predominantly moderate among participants (57.7%). Burnout emerged as a significant concern, with the majority experiencing moderate levels (94%). Similarly, trauma stress levels were predominantly moderate or low among participants, with only a small fraction reporting high levels (2.1%). Figure 1 depicts the participants’ physical activity and professional quality of life. The average value of compassion satisfaction was 38.7 ± 6.9, burnout 31.5 ± 4.7, and trauma stress was 24.2 ± 7.2.

Table 2 shows that nurses engaging in moderate physical activity had the highest overall professional quality of life (96.5 ± 13.1), as compared to high (94.4 ± 6.3) and low physical activity (92.1 ± 13.1), *p* < 0.001.

**Table 2:** Association between physical activity and professional quality of life

Variables	*N*	Compassion satisfaction, mean ± SD	Burnout, mean ± SD	Trauma stress, mean ± SD	Professional Quality of life
low	200	36.9 ± 7.2	31.0 ± 4.6	24.2 ± 6.9	92.1 ± 13.1
moderate	222	40.3 ± 6.3	32.0 ± 4.8	24.1 ± 7.5	96.5 ± 13.1
high	8	39.5 ± 5.0	31.4 ± 3.5	23.5 ± 6.9	94.4 ± 6.3
*p*-value		< 0.001	0.079	0.96	0.003

### Physical activity and sociodemographic, occupational, and health factors

Table 3 shows the association between physical activity and sociodemographic, occupational, and health-related factors. Age was significantly different across physical activity groups, with individuals in the moderate activity group being older on average 44.1 ± 7.1 years compared to those in the low (42.5 ± 7.3 years) and high activity groups (40.5 ± 5.9 years), *p* = 0.046. The percentage of nurses working 12-hour shifts was significantly higher in the high activity group (25.0%) compared to the low (5.0%) and moderate (5.0%) activity groups, *p* = 0.045. Additionally, a significant association was observed between COVID-19 infection and physical activity levels; 50.0% of individuals in the high-activity group reported no change in activity post-infection, compared to 35.1% in the moderate and 24.5% in the low-activity groups, *p* = 0.006. Self-monitoring devices were also significantly associated with physical activity level, with the moderate activity group having the highest usage (30.2%) and the high activity group having the least 12.5%, *p* = 0.029. Lastly, the perception of adequate physical activity was significantly different, with individuals in the high-activity group more likely to report satisfaction with their activity level (0.0% felt the need to do more) compared to the moderate (1.8%) and low-activity groups (5.5%), *p* = 0.035.

**Table 3:** Association between physical activity and sociodemographic, occupational, and health-related factors

Variables	Level	low	moderate	high	*p*-value
N		200	222	8	
Age, mean (SD)		42.5 (7.3)	44.1 (7.1)	40.5 (5.9)	0.046
Gender	Male	28 (14.0%)	46 (20.7%)	3 (37.5%)	0.069
	Female	172 (86.0%)	176 (79.3%)	5 (62.5%)	
Marital status	Single	46 (23.0%)	55 (24.8%)	3 (37.5%)	0.95
	Married	134 (67.0%)	145 (65.3%)	5 (62.5%)	
	Widowed	2 (1.0%)	2 (0.9%)	0 (0.0%)	
	Divorced	18 (9.0%)	20 (9.0%)	0 (0.0%)	
Region	Latin America	82 (41.0%)	121 (54.5%)	4 (50.0%)	0.23
	Middle East	20 (10.0%)	15 (6.8%)	0 (0.0%)	
	Southeast Asia	36 (18.0%)	32 (14.4%)	2 (25.0%)	
	South Asia	57 (28.5%)	46 (20.7%)	2 (25.0%)	
	Africa	5 (2.5%)	8 (3.6%)	0 (0.0%)	
Position	Bedside Nurses	164 (82.0%)	177 (79.7%)	8 (100.0%)	0.69
	Educator & specialist	17 (8.5%)	22 (9.9%)	0 (0.0%)	
	Administration	19 (9.5%)	23 (10.4%)	0 (0.0%)	
Education	Doctorate	2 (1.0%)	1 (0.5%)	0 (0.0%)	0.69
	Master’s degree	39 (19.5%)	44 (19.8%)	1 (12.5%)	
	Bachelor’s degree	155 (77.5%)	166 (74.8%)	7 (87.5%)	
	Associate’s degree/Diploma	4 (2.0%)	11 (5.0%)	0 (0.0%)	
Height (cm), mean (SD)		160.9 (8.7)	161.8 (7.8)	161.0 (7.8)	0.57
Weight (kg), mean (SD)		78.6 (60.1)	74.2 (13.4)	70.1 (9.5)	0.53
Regular Duty hours	12 h/ day	10 (5.0%)	11 (5.0%)	2 (25.0%)	0.045
	8 h/day	190 (95.0%)	211 (95.0%)	6 (75.0%)	
Types of Duty	Day duty only	63 (31.5%)	80 (36.0%)	1 (12.5%)	0.39
	Rotating shift with night duty	132 (66.0%)	133 (59.9%)	7 (87.5%)	
	Rotating shift without night duty	5 (2.5%)	9 (4.1%)	0 (0.0%)	
Are you infected with covid-19?	No	94 (47.0%)	100 (45.0%)	4 (50.0%)	0.90
	Yes	106 (53.0%)	122 (55.0%)	4 (50.0%)	
If yes, does it affect your physical activity level after covid-19 infection?	No	49 (24.5%)	78 (35.1%)	4 (50.0%)	0.006
	Yes, I feel easily exhausted after an infection	57 (28.5%)	44 (19.8%)	0 (0.0%)	
Did you use any self-monitoring tracking devices or smartphone applications to	No	161 (80.5%)	155 (69.8%)	7 (87.5%)	0.029
	Yes	39 (19.5%)	67 (30.2%)	1 (12.5%)	
Smoking status	Smoking daily	12 (6.0%)	11 (5.0%)	1 (12.5%)	0.39
	Occasional smoker	7 (3.5%)	8 (3.6%)	0 (0.0%)	
	Nonsmoker	175 (87.5%)	200 (90.1%)	6 (75.0%)	
	Former smoker	6 (3.0%)	3 (1.4%)	1 (12.5%)	
Do you feel that the current level of your physical activity is adequate for the main	No, I feel I need to do more for my physical health	11 (5.5%)	4 (1.8%)	0 (0.0%)	0.035
	Yes, it meets my needs for physical health	1 (0.5%)	4 (1.8%)	0 (0.0%)	

### Professional quality of life and sociodemographic, occupational, and health-related factors

The analysis (Table 4) revealed significant associations between compassion satisfaction, trauma stress, and various factors among nurses. Nurses from Latin America reported the highest compassion satisfaction (41.4 ± 6.0), while those from the Middle East had the lowest (34.1 ± 7.5), *p* < 0.001. Administrative staff had higher satisfaction (41.3 ± 5.9) than bedside nurses (38.5 ± 6.9), *p* = 0.027. Nurses working 8-hour shifts showed significantly higher compassion satisfaction (38.8 ± 6.8) than those working 12-hour shifts (35.8 ± 7.0), *p* = 0.037. Participants who had not contracted COVID-19 had higher compassion satisfaction (39.6 ± 6.6) than those who did (37.9 ± 7.0), *p* = 0.009. Furthermore, nurses engaging in moderate physical activity had the highest compassion satisfaction (40.3 ± 6.3), while those with low physical activity reported the lowest levels (36.9 ± 7.2), *p* < 0.001.

**Table 4:** Association between professional quality of life and sociodemographic, occupational, and health-related factors

Variables	*N*	Compassion satisfaction, mean ± SD	Burnout, mean ± SD	Trauma stress, mean ± SD	Professional Quality of life
**Gender**					
Male	77	38.4 ± 7.6	31.2 ± 5.4	24.4 ± 7.4	94.0 ± 14.2
Female	353	38.7 ± 6.7	31.6 ± 4.6	24.1 ± 7.2	94.5 ± 13.0
*p*-value		0.74	0.47	0.79	0.77
**Marital status**					
Single	104	39.8 ± 6.9	32.4 ± 4.8	23.4 ± 7.4	95.6 ± 12.7
Married	284	38.3 ± 6.6	31.2 ± 4.8	24.5 ± 7.1	94.0 ± 13.5
Widowed	4	38.3 ± 8.7	33.8 ± 1.9	27.3 ± 4.6	99.3 ± 5.0
Divorced	38	38.6 ± 8.5	31.5 ± 4.2	23.3 ± 7.8	93.4 ± 12.9
*p*-value		0.30	0.16	0.40	0.61
**Region**					
Latin America	207	41.4 ± 6.0	31.7 ± 5.2	23.2 ± 7.3	96.3 ± 13.9
Middle East	35	34.1 ± 7.5	31.3 ± 3.1	27.5 ± 5.7	92.8 ± 10.9
Southeast Asia	70	36.5 ± 7.0	32.1 ± 4.0	23.3 ± 7.1	91.8 ± 11.1
South Asia	105	36.4 ± 6.1	31.1 ± 4.9	25.6 ± 7.1	93.1 ± 13.9
Africa	13	39.0 ± 6.5	29.7 ± 2.8	23.6 ± 7.0	92.3 ± 7.9
*p*-value		< 0.001	0.35	0.003	0.061
**Position in HMC**					
Bedside Nurse	349	38.5 ± 6.9	31.5 ± 4.9	24.1 ± 7.3	94.1 ± 13.6
Educator or Specialist	39	37.8 ± 7.4	31.9 ± 4.2	25.5 ± 7.7	95.3 ± 11.9
Administration	42	41.3 ± 5.9	31.4 ± 3.9	23.2 ± 5.7	96.0 ± 10.9
*p*-value		0.027	0.87	0.33	0.63
**Highest level of education**					
Doctorate	3	42.0 ± 1.0	34.7 ± 3.1	26.3 ± 3.2	103.0 ± 4.4
Master’s degree	84	38.8 ± 8.2	31.8 ± 5.3	24.3 ± 7.6	95.0 ± 14.4
Bachelor’s degree	328	38.7 ± 6.5	31.5 ± 4.6	24.2 ± 7.2	94.3 ± 13.1
Associate’s degree/Diploma	15	37.4 ± 6.8	30.9 ± 3.4	23.1 ± 6.0	91.3 ± 9.5
*p*-value		0.74	0.58	0.88	0.52
**Working hours**					
12 h/ day	23	35.8 ± 7.0	30.8 ± 4.0	24.0 ± 7.2	90.6 ± 10.6
8 h/day	407	38.8 ± 6.8	31.6 ± 4.8	24.2 ± 7.2	94.6 ± 13.3
*p*-value		0.037	0.43	0.93	0.16
**Type of duty**					
Day duty only	144	39.0 ± 7.0	31.5 ± 4.5	24.2 ± 7.1	94.8 ± 13.1
Rotating shift with night duty	272	38.6 ± 6.9	31.6 ± 4.9	24.1 ± 7.2	94.2 ± 13.3
Rotating shift without night duty	14	36.6 ± 5.6	31.5 ± 4.5	25.4 ± 7.7	93.6 ± 11.9
*p*-value		0.45	0.99	0.78	0.90
**Covid Infection**					
No	198	39.6 ± 6.6	31.3 ± 4.9	23.3 ± 7.2	94.2 ± 14.0
Yes	232	37.9 ± 7.0	31.8 ± 4.6	24.9 ± 7.1	94.6 ± 12.5
*p*-value		0.009	0.26	0.017	0.72
**If yes does it affect your physical activity?**					
No	131	39.0 ± 6.7	31.5 ± 5.0	23.5 ± 7.3	94.1 ± 13.7
Yes, I feel easily exhausted after an infection	101	36.4 ± 7.1	32.2 ± 4.0	26.8 ± 6.4	95.3 ± 10.8
*p*-value		0.004	0.26	< 0.001	0.44
**Self-Monitoring**					
No	323	38.7 ± 6.9	31.4 ± 4.7	24.2 ± 7.2	94.3 ± 13.3
Yes	107	38.7 ± 6.8	32.0 ± 4.7	24.1 ± 7.2	94.8 ± 12.8
*p*-value		0.92	0.22	0.85	0.69
**Smoking Status**					
Smoking daily	24	37.7 ± 8.5	31.2 ± 6.8	25.7 ± 8.4	94.6 ± 19.0
Occasional smoker	15	38.9 ± 9.6	29.6 ± 5.4	22.9 ± 8.0	91.5 ± 17.4
Nonsmoker	381	38.8 ± 6.7	31.6 ± 4.6	24.1 ± 7.1	94.6 ± 12.7
Former smoker	10	36.7 ± 5.1	31.5 ± 4.0	23.9 ± 5.4	92.1 ± 9.8
*p*-value		0.69	0.42	0.67	0.78
**Current level of physical activity**					
No, I feel I need to do more for my physical health	15	36.7 ± 7.7	31.7 ± 3.8	26.5 ± 5.7	94.9 ± 10.0
Yes, it meets my needs for physical health	5	37.4 ± 10.0	32.6 ± 5.6	27.2 ± 10.8	97.2 ± 12.4
*p*-value		0.87	0.68	0.86	0.67

Burnout showed no significant associations with socio-demographics, health-related factors, or physical activity. Trauma stress was significantly associated with nationality, COVID-19 infection, and the changes in physical activity levels following infection. Middle Eastern nurses had the highest trauma stress levels (27.5 ± 5.7), while those from Latin America had the lowest (23.2 ± 7.3), *p* = 0.003. Nurses who had contracted COVID-19 had significantly higher trauma stress (24.9 ± 7.1) compared to those who had not (23.3 ± 7.2), *p* = 0.017. Those who reported feeling easily exhausted after COVID-19 infection had significantly higher trauma stress (26.8 ± 6.4) than those who did not experience exhaustion (23.5 ± 7.3), *p* < 0.001.

## Discussion

This study investigates the association between physical activity and professional quality of life (ProQOL) among nurses in Qatar, considering various sociodemographic, occupational, and health-related factors. The findings align with existing literature but highlight unique challenges in the Qatari context, contributing to a deeper understanding of the relationship between physical activity and well-being in healthcare professionals.

The study identified that nearly half of the participants reported low physical activity levels, while most others engaged in moderate physical activity. This trend is consistent with previous research indicating that healthcare workers, including nurses, often struggle to meet recommended physical activity guidelines due to occupational physical activity demands, fatigue, and long working hours [21, 22]. Globally, healthcare workers exhibit reduced physical activity levels despite being in professions advocating health promotion [11]. The relatively low engagement in high levels of physical activity among the study participants aligns with findings from previous studies in the Middle East, where nurses face cultural and systemic barriers to adopting active lifestyles [5].

Technology-based self-monitoring has been shown to foster health-promoting behaviors by enhancing motivation and awareness [23]. However, the study found that self-monitoring device usage was lower in the high physical activity group, suggesting that intrinsic motivation or other environmental factors might play a more significant role in sustaining high levels of physical activity in some cases [24].

The findings of this study align with existing literature emphasizing the positive association between physical activity and professional quality of life among nurses. Nurses engaging in moderate physical activity demonstrated the highest ProQoL scores, consistent with research suggesting that moderate activity optimally balances physical and psychological benefits, such as improved mood, stress reduction, and resilience to burnout [25]. Recent studies have reported that moderate-intensity exercise enhances emotional well-being and reduces occupational stress [26]. Interestingly, high physical activity showed slightly lower ProQoL scores, potentially reflecting challenges like overexertion or insufficient recovery time, a phenomenon also noted in earlier research [27]. Conversely, low physical activity, associated with the lowest ProQoL scores, highlights the risks of inactivity, including increased burnout and reduced job satisfaction [25]. These findings underline the importance of promoting moderate physical activity levels to enhance ProQoL among nurses, addressing physical and mental health needs critical to their demanding roles.

While considering the subdomains of ProQoL, the results confirm that moderate physical activity correlates with higher levels of compassion satisfaction, an essential aspect of ProQoL Nurses engaging in moderate physical activity reported the highest compassion satisfaction, reinforcing the notion that maintaining a healthy balance of physical activity contributes to professional fulfillment [28]. In contrast to other studies that associate Physical activity with reduced burnout, this research found no significant relationship between burnout and physical activity levels [29]. This discrepancy may suggest that factors beyond physical activity, such as organizational support, emotional intelligence, and workload, play a more pivotal role in determining burnout levels [30]. Additionally, Qatar’s unique work environment and cultural context may influence burnout differently than in Western settings [31].

This study highlights significant associations between nurses’ physical activity levels and sociodemographic characteristics, particularly age. Nurses engaging in moderate physical activity were slightly older (44.1 ± 7.1 years) compared to those in low (42.5 ± 7.3 years) or high (40.5 ± 5.9 years) activity groups. This aligns with prior research suggesting that middle-aged individuals are more likely to adopt exercise routines as part of health maintenance strategies. In contrast, younger individuals may have the capacity for more intense physical activity due to fewer physical and professional constraints [32].

Gender differences in physical activity levels were consistent with global patterns. Male nurses, although a minority in the sample, were more likely to engage in higher physical activity levels than females. This trend is supported by studies indicating that men generally participate more in vigorous physical activities than women, often influenced by cultural and social norms [33]​. However, the predominantly female composition of the nursing profession might skew this observation, underscoring the need for broader investigations into gender-specific barriers to physical activity in healthcare settings [34].

Work-related factors, particularly shift patterns, also influenced physical activity levels. Nurses on rotating shifts with night duties reported lower physical activity levels than those on fixed day shifts or 12-hour schedules. This finding corroborates studies showing the negative association of irregular work hours on physical activity, often due to disrupted circadian rhythms and fatigue [35]​. Interestingly, participants working 12-hour shifts were more likely to report high physical activity levels, potentially reflecting the physically demanding nature of these shifts. This observation aligns with studies linking physically intensive work environments to higher energy expenditures during duty hours​ [36].

Professional quality of life, encompassing compassion satisfaction, burnout, and trauma stress, demonstrated significant associations with sociodemographic and occupational variables, reinforcing findings from previous studies. Compassion satisfaction was highest among nurses from Latin America and those in administrative roles. This may be attributed to cultural differences in workplace dynamics aligning with research suggesting that supportive work environments and reduced direct patient interaction enhance job satisfaction [37]​.

Shift length also influenced compassion satisfaction, with nurses working 8-hour shifts reporting higher levels than those on 12-hour schedules. This finding is consistent with studies indicating that shorter shifts improve work-life balance and reduce occupational stress, ultimately enhancing compassion [38, 39].

Trauma stress was significantly associated with nationality and COVID-19 infection. Nurses from the Middle East exhibited the highest levels of trauma-related stress, whereas those from Latin America reported the lowest. These findings align with previous studies, highlighting the intricate interplay of cultural, social, and systemic factors that shape nurses’ mental health [40]. The elevated trauma stress among Middle Eastern nurses can likely be attributed to the compounded effects of cultural stigmas surrounding mental health and societal norms that may discourage open discussions about psychological distress [41].

Furthermore, nurses who contracted COVID-19 experienced higher trauma stress levels, particularly those reporting post-infection fatigue, highlighting the lasting psychological impact of the pandemic on healthcare workers. Similar findings have been documented in studies exploring the long-term effects of COVID-19 on frontline workers, emphasizing the need for ongoing psychological support​ [42].

During the COVID-19 pandemic, the dynamics of physical activity significantly affected nurses’ overall well-being, as explored in studies by Nashwan et al. (2021). The PROTECTOR study highlights that physical inactivity among nurses contributed to heightened stress, poor sleep quality, and diminished overall quality of life, exacerbated by the unique demands of the pandemic. Nurses, who were often constrained by long hours and high-stress environments, faced substantial challenges in maintaining regular physical activity [43]. Another study observed that physical activity positively correlated with lower turnover intentions, suggesting that moderate physical activity could buffer the adverse effects of pandemic-induced occupational stress [44]. The findings underscore the need for institutional strategies to facilitate accessible and sustainable physical activity programs, particularly during health crises, to bolster resilience and mitigate mental health risks for healthcare professionals.

## Limitation

The cross-sectional study design limits the ability to establish a causal relationship between physical activity and ProQoL. While associations were identified, the temporal relationships between these variables remain unclear. The reliance on self-reported data for assessing physical activity and professional quality of life introduces the possibility of reporting bias or inaccuracies, as participants may overestimate or underestimate their behaviors and perceptions. Furthermore, cultural and workplace dynamics specific to Qatar may influence physical activity and professional quality of life in ways that differ from other contexts, posing challenges to the applicability of these findings globally. Lastly, potential confounding factors such as dietary habits, mental health status, and personal stressors were not controlled in this study, which may have influenced the observed relationships. Future research addressing these limitations is recommended to validate and expand upon the findings presented here.

## Conclusion

This study highlights the critical role of moderate physical activity in improving nurses’ professional quality of life, specifically by fostering compassion satisfaction. The findings emphasize that while moderate PA optimally balances physical and psychological benefits, other factors may influence burnout and trauma stress, warranting further exploration. These insights underscore the need for targeted strategies to promote physical activity within healthcare settings, such as incorporating wellness programs and providing accessible PA opportunities during work shifts. Recognizing the unique challenges nurses face in Qatar, culturally sensitive interventions are essential to overcoming barriers to active lifestyles. Ultimately, fostering moderate physical activity among nurses not only benefits their well-being but also enhances their capacity to deliver quality patients.

## Data Availability

The data that support the findings of this study are available from the corresponding author upon reasonable request.
